# RISE-Vac—Co-Production of Vaccine Education Materials with Persons Living in Prison

**DOI:** 10.3201/eid3013.230812

**Published:** 2024-04

**Authors:** Femi Laryea-Adekimi, Jemima D’Arcy, Angela Bardelli, Aurélie Mieuset, Vlad Busmachiu, Irina Barbiros, Fadi Meroueh, Svetlana Doltu, Niall Walsh, Paula Harriott, Lara Tavoschi, Emma Plugge, Alicia Roselló

**Affiliations:** Prison Reform Trust, London, UK (F. Laryea-Adekimi, P. Harriott);; United Kingdom Health Security Agency, London (J. D’Arcy, E. Plugge, A. Roselló);; University of Pisa, Pisa, Italy (A. Bardelli, L. Tavoschi);; Centre Pénitentiaire, Villeneuve-lès-Maguelone, France (A. Mieuset);; National Administration of Penitentiaries, Chișinău, Moldova (V. Busmachiu, I. Barbiros);; University Hospital of Montpellier, Montpellier, France (F. Meroueh);; State University of Medicine and Pharmacy “Nicolae Testemiţanu,” Chișinău (S. Doltu);; Pathways Centre, Dublin, Ireland (N. Walsh);; University of Southampton, Southampton, UK (E. Plugge)

**Keywords:** co-production, educational materials, vaccination, immunization, prison, people living in prison, prisoner, inmate, jail, vaccine learning, vaccine-preventable diseases, United Kingdom, France, Moldova

## Abstract

Increasing vaccination knowledge is effective in addressing hesitancy and is particularly important in populations deprived of liberty who may not routinely have access to health information, ensuring health equity. RISE-Vac is a European Union–funded project aiming to promote vaccine literacy, offer, and uptake in prisons in Europe. We consulted persons living in prisons in the United Kingdom (through the Prisoner Policy Network), France, and Moldova to determine their vaccination knowledge gaps, the information they would like to receive, and how they would like to receive it. We received 344 responses: 224 from the United Kingdom, 70 from France, and 50 from Moldova. Participants were particularly interested in learning about the effectiveness, side effects, and manufacturing of vaccines. Their responses guided the development of educational materials, including a brochure that will be piloted in prisons in Europe. Persons with experience of imprisonment were involved at every stage of this project.

Incarcerated persons have a higher prevalence of infectious diseases than the general community (*1*). This disparity can be linked to many factors, including contextual factors of the prison setting, such as overcrowding, delays in diagnosis and treatment, and high population turnover (*2*), and population characteristics, including higher prevalence of smoking cigarettes and engaging in commercial sex work (*3*,*4*). However, some diseases with higher prevalence among prison populations, such as human papillomavirus, influenza, and viral hepatitis (*5*,*6*), can be prevented through vaccination. Vaccination remains one of the most cost-effective public health interventions in the community; in the prison context, vaccination could help control infectious disease transmission and outbreaks, reducing illness and death among persons living in prison as well as protecting prison staff and the rest of the community (*7*,*8*). However, global data on vaccination in prisons is inadequate; a recent study examining COVID-19 vaccination rates found that, in the 6 countries that had prison vaccination data, rates were lower than for the general population (*9*).

RISE-Vac (Reaching the hard-to-reach: increasing access and vaccine uptake among prison populations in Europe) is a 3-year project funded by the European Union’s Health Programme (*10*). RISE-Vac is led by the University of Pisa in Pisa, Italy, and consists of 8 further consortium partners based in Cyprus, France, Germany, Italy, Moldova, and the United Kingdom. The project seeks to increase vaccine access and uptake in prison populations across Europe. In this context, prisons include both pretrial and postadjudication facilities. One such intervention is the development and implementation of educational tools aimed at increasing vaccine knowledge in persons living in prison. Educational interventions, including knowledge dissemination through posters, pamphlets, or brochures, have previously been implemented in the prison context and have been shown to increase vaccine literacy and uptake of screening programs (*11*). Although the COVID-19 pandemic raised awareness of the importance of vaccination in controlling infectious diseases and the problems of vaccine hesitancy, this project is not focusing on a specific vaccine but vaccination in general, acknowledging that acceptability differs according to the vaccine and the infection.

Persons who have been or are currently imprisoned are too often left out of the development of interventions targeting prison populations (*12*). The perspective of those who have this direct experience is likely to be key to increasing the effectiveness and relevance of these interventions. Although robust evidence for engagement of incarcerated populations in co-production is lacking, the World Health Organization advocates for this approach in patient populations, stating that “resources may be better used if they are aligned with patients’ priorities” (*13*). The RISE-Vac project partnered with persons who had been imprisoned but were now working for the Prison Reform Trust (PRT), a charity in England, to co-produce educational tools on vaccination for persons currently imprisoned across Europe. In this article, we present this co-production methodology and the resulting educational tool, developed with the input of persons who have been imprisoned and those who are currently imprisoned in the United Kingdom, France, and Moldova.

## Methods

To direct the development of the educational materials, in early 2022, the United Kingdom Health Security Agency (UKHSA), the United Kingdom partner, set up an advisory group consisting of experts in the field of prison health with knowledge of vaccination in prisons, experts in developing educational materials for persons living in prison, and persons with lived experience of imprisonment from across Europe (Appendix). We aimed for a minimum of 1 person per country participating in RISE-Vac to ensure the context of all participating countries was represented; each country did not provide an expert in each area, but we ensured that the advisory group as a whole had experts in all relevant areas. 

The PRT has a network called the Prisoner Policy Network (PPN) comprising >700 persons living in all prisons across the 4 countries of the United Kingdom and persons now back in the community. PPN membership is open to anyone who has been in or is currently in prison in the United Kingdom. During the last 6 months of 2022, PRT consulted members of the PPN to obtain their views on vaccines and determine what further information they would like to receive about vaccination. All PPN members were eligible to participate regardless of their vaccination status or views. Integrating feedback from the advisory group, PRT produced a set of 7 questions to draw out the views of those living in prison. PRT piloted those questions in His Majesty’s Prison (HMP) Rye Hill with a group of 10 incarcerated persons who extended this pilot to their social network. PRT received 30 written responses from HMP Rye Hill and oral feedback on the questions asked. In response, PRT adjusted the order of the questions and included 2 additional questions regarding family views of vaccines (Table).

**Table:** Consultation questions to elicit views on vaccination among those living in prison or with lived experience of prison as part of the European Union’s RISE-Vac project*

Question no.	Question
1	Have you had any vaccines in your life?
2	Tell us your opinion about vaccination and vaccines in general (not only COVID-19 vaccines).
3	What do you already know about vaccines?
4	What more would you like to know about vaccines?
5	Are you confident you have enough reliable information about vaccines?
6	Who do/would you trust to give you that reliable information?
7	What is the opinion of your friends and family about vaccination and vaccines in general (not only COVID-19 vaccines)?
8	Does the opinion of your family and friends about vaccination matter in your decision to vaccinate? If so, how?
9	How would you like to receive the information you want about vaccines? (verbally, short leaflet, detailed manual, video, audio, discussion groups)

*RISE-Vac, Reaching the hard-to-reach: increasing access and vaccine uptake among prison populations in Europe.

In early 2023, PRT set out to consult persons from all RISE-Vac partner countries with the questions translated into Romanian, French, and Italian. The RISE-Vac leads in Moldova and France distributed the translated questions to all persons living in prison in 2 prisons in each of those countries. Data were not collected on the demographics of those who responded. At this time PRT ran a focus group in the community in the United Kingdom consisting of 4 persons who had been imprisoned and who identified as vaccine hesitant and 1 moderator with experience of incarceration. The same questions were asked to these participants.

All written responses were translated into English if necessary, collated, and analyzed by using thematic analysis to determine the key concepts (*14*). After familiarization with the data, initial codes were developed (open coding) by a person from PRT and 2 members of UKHSA who had with expertise in qualitative methods. The data were coded independently and then agreed in an initial meeting and subsequently refined by a series of discussions. Those discussions led to the organization of the codes into conceptual categories, themes, and subthemes. This process guided the development of a brochure designed to be distributed in all prisons in Europe to enable vaccine learning.

The RISE-Vac project received ethics approval from the Committee on Bioethics of the University of Pisa (approval no. 0049433/2022). This specific piece of work did not require ethics approval because it was a consultation exercise as part of a health improvement initiative. No personal identifiers (e.g., demographic information) were recorded on the response form. No incentives for participation were provided.

## Results

PRT received 224 responses from incarcerated persons in the United Kingdom, 50 from Moldova, and 70 from France. Responses were received from both male and female prisons, but data on respondent demographics were not collected at an individual level. It was not possible to establish how many persons had been approached and therefore the number of persons who refused to participate.

Although this convenience sample was not selected on the basis of vaccination status, all respondents had received >1 vaccine in their lifetime. The key themes were common across the 3 participating countries: views of vaccination, prior knowledge about vaccines, areas of appetite for learning, availability of reliable information, and preferred mechanism for information sharing.

### Views on Vaccination

Despite a generally positive view of vaccines from the United Kingdom respondents, some were not as convinced about the benefits of vaccines as others:

“They’re not 100% but they help persons and save lives.”
“I have a certain amount of trust in vaccines, but you can never be 100% about them as after all it is a foreign body going into your own body.”

Similarly, some respondents in Moldova expressed doubts about the effectiveness of vaccines:

“My opinion is that the vaccine is not the best method for protecting your own health.”
“All vaccines do not inspire confidence in me. My opinion is that these vaccines are tests for the population.”

In France, respondents were more positive about vaccines in general but were particularly skeptical about COVID-19 vaccines:

“I believe in traditional vaccines, because they have been researched for years. I have no confidence in COVID-19 vaccines; how come we haven't been able to find vaccines against AIDS since 1985, and just like that we found vaccines for COVID in 2 years.”
“It could be good for preventing diseases but the anti-vax discourse also has good arguments.”

In the focus group, participants expressed skepticism about the rapid production of the COVID-19 vaccines and the perceived pressure put on the public to take the vaccines. They were more comfortable with established vaccines including vaccines required for tropical diseases when traveling.

### Prior Knowledge about Vaccines

Respondents expressed a desire and a need for more information than the basic knowledge they already had regarding vaccines. In the United Kingdom, incarcerated persons reported having the following information about vaccines:

“Nothing scientific really, I try to pick up on any advice and guidance out there. But it can be confusing or misleading.”
“They build or prepare your immune system to effectively fight the virus, allegedly.”

Respondents in Moldova expressed these thoughts:

“Thanks to them, I can get immunity to diseases.”
“We practically do not have any information to confirm that these vaccines help.”

In France, some respondents said they didn’t know anything, or only very little. However, others said that they were aware vaccines aided with immunity and protection from diseases.

### Areas of Appetite for Learning

When asked what additional information they wanted to receive about vaccines, many respondents in the United Kingdom felt they already had enough information to make decisions on vaccination. However, most wanted access to more information, particularly about side effects of vaccines:

“[Nothing] especially. I think I know the basics.”
“Possible side effects. Effectiveness against different viruses. Basic make up and formulation.”

Respondents in Moldova repeatedly asked for detailed information about vaccines:

“Detailed information (where the vaccine is produced, in which laboratory, the consistency of the vaccine)”
“Everything possible: vaccine types, possible side effects, why do I need them?”

In contrast many respondents in France did not want any more information. However, some participants asked for more information on vaccine efficacy, vaccine production processes, contents of vaccines, and side effects of vaccines.

### Availability of Reliable Information

Many respondents in the United Kingdom felt they did not have access to reliable information while in prison. However, most respondents felt they already had enough information to make a decision.

Most respondents in Moldova did not feel they had enough information to make an informed decision. This sentiment was echoed in France, where most respondents felt they did not have enough information.

### Trusted Source for Reliable Information

Most incarcerated persons expressed that they would trust medical professionals to deliver vaccine information more than other sources, such as custodial staff. The respondents’ thoughts regarding family views varied across the countries consulted. In Moldova, respondents’ families’ views emerged as an important factor affecting their decision, in contrast to respondents from the United Kingdom and France, who did not cite family views as important factors.

### Preferred Mechanism for Information Sharing

In the United Kingdom, a short leaflet was the delivery mode most incarcerated persons preferred, followed by verbal delivery, then video. In Moldova, discussion groups with medical professionals were the most favored delivery mechanisms, followed by a detailed manual. In France, verbal delivery was most popular, although a short leaflet and video also were favored mechanisms.

### Materials Developed

In line with the findings of the consultation, we produced an illustrated brochure (Appendix). This initial draft of the brochure is undergoing review by the international advisory committee and UKHSA vaccination experts before wider dissemination.

## Discussion

The results of the consultation demonstrate the desire from incarcerated persons to be equipped with accurate information to make informed decisions about vaccines. Many reported the lack of information they have access to in prison and felt limited by this lack. We were in a position to remedy this by producing materials that can be made accessible to persons living in prison and thereby encourage vaccine uptake in prisons.

Incarcerated persons, those who have been imprisoned, or both were involved at all stages of development of this brochure, including the leadership of the work, consultation, and drafting of the brochure and this article. The advisory group and immunization experts provided support, ensuring the robustness of content from a scientific perspective. This true co-development approach is necessary for the development of relevant and ethically developed materials. Although this approach is not yet widely piloted, we hope that the process of development will ensure that incarcerated persons will engage with the materials that have been informed by their peers. This aspect is important given that a recent scoping review examining COVID-19 vaccination in prisons found high levels of vaccine hesitancy among incarcerated persons and that a lack of educational materials about vaccines increased any concerns, potentially leading to feelings of apathy or beliefs in conspiracy theories (*15*). The impact of these educational resources will be evaluated during the RISE-Vac study by using a questionnaire survey examining knowledge, attitudes, and behaviors before and 1–3 months after implementation. Longer-term and more extensive evaluation is not possible given restraints on study resources and timeframes.

One limitation of this study is that the consultation process may have been exclusionary to certain cohorts. By using a written format, we may have excluded those with low literacy. We also may have discouraged some persons with negative views of vaccines to participate just by asking them to respond on the subject at a time where some sensitivity regarding vaccination choices exists, especially in prison. In addition, whereas PPN in the United Kingdom does include women and younger incarcerated persons, its members are overwhelmingly adult men. Therefore, the needs of those with low literacy, those who are vaccine resistant, women, and younger incarcerated persons may have not been captured. Because we did not collect data on the demographic characteristics of respondents and nonrespondents, we cannot be certain about whose views were not gathered. Furthermore, we were unable to collect denominator data and therefore cannot be sure of the response rate, nor how that rate differed by demographic characteristics.

All materials used in this study will be piloted and translated into the languages of all RISE-Vac partner countries and additional languages as relevant to their prison context. In addition, a video animation covering the brochure content will be developed and dubbed. These materials then will be disseminated across Europe through RISE-Vac. Study funding limits meant that there were not resources to develop materials to support discussion groups with medical professionals (the preferred option of respondents) but this aim should be considered as a priority in the future. Similarly, this work demonstrates that participants might benefit from information about specific vaccines, and although it has not been possible to undertake within RISE-Vac, this focus should be a key development for the future. Creators of such materials will be able to build on this work, whether in response to pandemics and outbreaks or for routine vaccination.

Through this consultation process, we recognized a need for vaccine information in prison; incarcerated persons should have access to this resource to make informed decisions. Prisons do not exist outside of society, and so prison healthcare is connected to and impacts public health; prison health is public health (*16*). We have aimed to address the educational and information needs of incarcerated persons about vaccination to enable them to make informed decisions, ultimately improving vaccine uptake in prisons and aiding society as a whole to improve protection from vaccine-preventable diseases.

Appendix Additional information about RISE-Vac: co-production of vaccine education materials with persons living in prison.
