# Supplementary material for: RISE-Vac—Co-Production of Vaccine Education Materials with Persons Living in Prison
Source: Emerg Infect Dis. 2024 Apr;30(Suppl 1):S56–61. doi: 10.3201/eid3013.230812 (PMC10986827; doi:10.3201/eid3013.230812)
Supplement: Appendix — Additional information about RISE-Vac: co-production of vaccine education materials with persons living in prison. [file 23-0812-Techapp-s1.pdf]

Article DOI: https://doi.org/10.3201/eid3013.230812

RISE-Vac—Co-production of Vaccine Education Materials with Persons Living in Prison

Appendix

Terms of Reference and Composition of the WP6 Advisory Group on Improving Vaccination Knowledge of Persons Living in Prison

Purpose of the Advisory Group

This advisory group is created to support the work of the RISE-Vac study, specifically in relation to WP6 (i.e., work package no. 6 of the RISE-Vac project, under which this specific piece of work was conducted), in the aim of increasing vaccine literacy for persons in prison.

The group shall provide advice on matters relating to the development of audiovisual materials for persons living in prison. These may include animations, other types of videos, written materials and audio materials.

The group are expected to undertake the following:

- Advise on the learning outcomes for the materials to be produced
- Advise on the format of materials
- Evaluate the first and subsequent drafts of learning materials developed in English
- Give context-specific advice, so as to guide the development of materials that can be relevant across European prisons

The advisory group will make recommendations and the decision to accept these recommendations will ultimately be made by the lead partner of WP6, UKHSA.

Membership

The advisory group will include representation from

- experts in the field of prison health with knowledge of vaccination in prisons,
- experts in developing educational materials for persons living in prison, and
- persons with lived experience of imprisonment.

We aim for a minimum of 1 person per country participating in RISE-Vac to ensure the context of all participating countries is represented; however due to time constraints this may not be possible at every meeting. There should be a maximum of 12 members. Members are expected to hold office for the duration of the project up until May 2024. If leaving the project before this, they will be requested to select someone to replace them.

As this is an advisory group, there is no minimum number for the meetings to be quorate.

Responsibilities of Members

The membership of the group will commit to:

- attending all scheduled Steering Committee meetings and actively and respectfully contributing to plans and discussions
- commenting on drafts of educational materials
- making timely recommendations and taking action so as to not hold up work
- active participation with the group within and outside of scheduled meetings
- reading of the minutes and all papers in advance any meetings
- open and honest discussions

Communication

Advisory group members will be addressed by e-mail. They will be notified in advance of meetings and agenda and minutes from previous meetings (if relevant) will be circulated. Any inaccuracies on minutes circulated should be raised directly by the advisory group by e-mail.

The minutes will be shared with the project coordinator, University of Pisa, for governance reasons.

Frequency, Location, and Management of Meetings

Advisory group meetings will be scheduled every 1–3 months over teleconference, as required, until the development of the materials is completed. If required subgroup meetings will be arranged outside of these times at a time convenient to subgroup members.

Appendix Table. Advisory Group Composition

Country	Position	Organization
Cyprus	Manager	Nongovernmental organization (NGO)
Cyprus	Deputy director	National Prison Service
France	Doctor working in prisons	Health without Barriers
Germany	Researcher	National NGO
Italy	Prison Researcher	University of Pisa
Italy	Lived Experience	NGO
Ireland*	Centre Manager, Lived experience	Ireland
Moldova	Head of Section	National NGO
Moldova	Deputy director	National Prison Service
Moldova	Deputy director	National Prison Service
Moldova	Head of medical department	National Prison Service
Moldova	Doctor working in prisons	National Prison Service
Moldova	Member	National NGO
UK	Senior Substance Misuse Integration Manager	NHS England and NHS Improvement
UK	Head of Prisoner Engagement, Lived experience	National NGO
UK	Immunization publications manager	UKHSA
UK	Infectious disease specialist	Public health Wales

*Chair of the group.

Brochure

The following pages show the first draft of the brochure created after the work described in the article. The draft is currently under review by experts in vaccination communication before wider dissemination among persons living in prison.

VACCINATION IN PRISON

Helping you make informed decisions

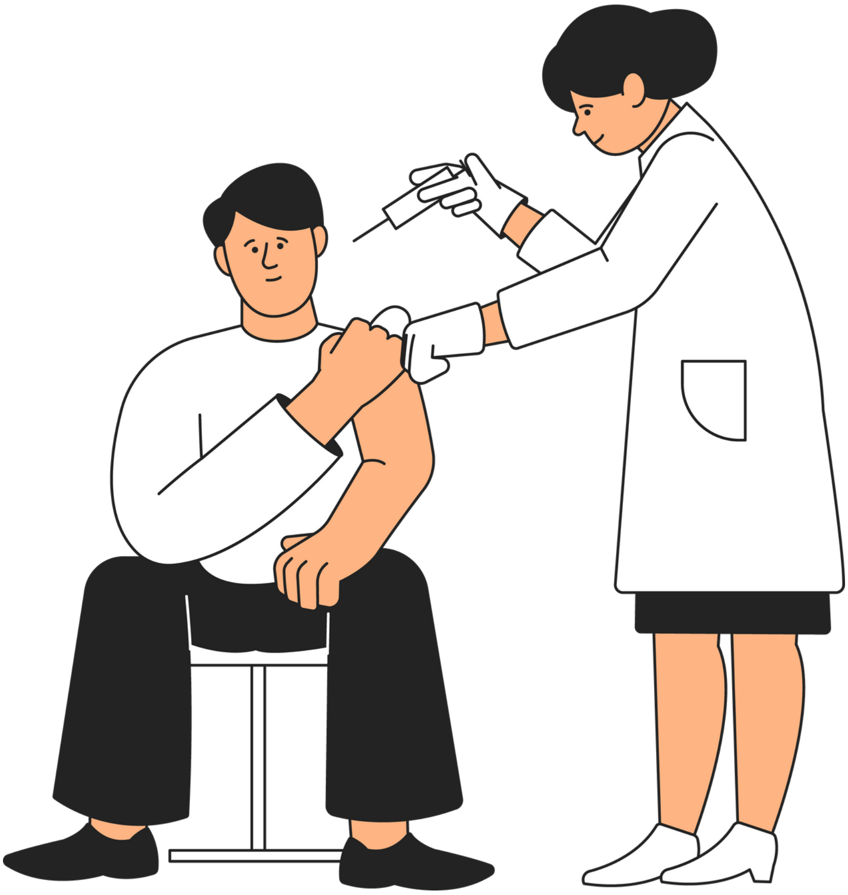

RISE-Vac

FRANKFURT UNIVERSITY OF APPLIED SCIENCES

CHU MONTPELLIER
CENTRE HOSPITALIER UNIVERSITAIRE

UK Health Security Agency

Ospedale San Paolo Polo Universitario
Sistema Socio Sanitario
Regione Lombardia
ASST Santi Paolo e Carlo

CYPRUS NATIONAL ADDICTIONS AUTHORITY

IN SUPREMÆ DIGNITATIS 1343
UNIVERSITÀ DI PISA

HEALTH WITHOUT BARRIERS

CYPRUS PRISONS DEPARTMENT 1960

DRAFT

Co-funded by the Health Programme of the European Union

This brochure was funded by the European Union's Health Programme (2014-2020). The content of this brochure represents the views of the author only and is his/her sole responsibility; it cannot be considered to reflect the views of the European Commission and/or the European Health and Digital Executive Agency (HaDEA) or any other body of the European Union. The European Commission and the Agency do not accept any responsibility for use that may be made of the information it contains.

PROVIDING WHAT YOU ASKED FOR

It is important to be **informed** when making a decision about medical care, and in prison it can be very difficult to get the information you want. This brochure aims to inform people living in prison about vaccines so that **you** can make informed decisions about vaccination. Hundreds of people living in prisons across Europe were asked what information they would like to receive about vaccines, and we have included the information they requested in this document.

Our project is called **RISE-Vac**. RISE-Vac is a European project aiming to increase knowledge about vaccines in prisons across Europe and is made up of nine different institutions (universities, prisons, medical centres, national public health organisations) across Europe. You can find details of all these organisations on the last page of this document. All of the work, including asking people living in prisons what they wanted to receive, was funded by the European Commission; former prisoners have been deeply involved in the co-production of this brochure and we have drawn on their understanding of prison to make sure that the information is presented in a way that is relevant.

DRAFT

KEY FACTS

1. Vaccines are a very effective way to prevent infections and have been used for over 200 years. Each year **vaccines prevent millions of deaths.**

2. The prison environment often makes it easy for **infection to spread quickly**. Other factors such as poor general health before prison also mean that people living in prison can be more vulnerable to catching infections. That's why it is important for you to have access to vaccines and be informed about vaccination.

3. Vaccines go through a **very careful production process** before being approved for medical use, this includes checking that they are safe and effective.

DRAFT

KEY FACTS

4

Vaccines **teach your immune system** to protect your body from diseases in a safer way than by catching the disease itself. Most vaccines offer a very high level of protection against infections that can be severe or deadly if you catch them.

5

As with any medicine, there can be side effects from vaccination, but these are **usually mild** and rarely have as much impact as actually catching the disease would.

6

Not everyone can be vaccinated. By vaccinating yourself you contribute to **protecting others** from infections too.

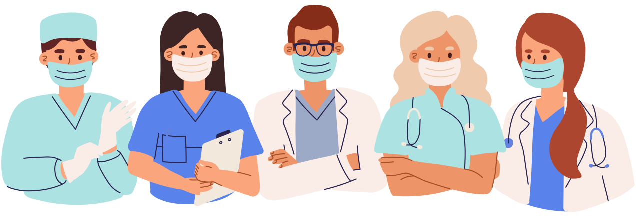

If you have any questions about vaccination, you can ask healthcare staff working in the prison.

DRAFT

WHAT ARE THE BENEFITS OF VACCINATION?

Being vaccinated against the most common infectious diseases in prison helps you and others stay healthy.

Vaccines are a very effective way to prevent infections, each year vaccines prevent millions of deaths. Being vaccinated against the most common infectious diseases in prison will help you stay healthy. It is much safer for your body to learn how to fight a disease through vaccination, than by catching the disease and treating it.

Not everyone can be vaccinated. For example some of those who are being treated for cancer cannot receive certain vaccines. By vaccinating yourself, you contribute to protecting others from infections too.

The more people in your prison who are vaccinated, the better the **protection** will be as the disease will have less chance to spread widely.

DRAFT

WHY IS VACCINATION SO IMPORTANT IN PRISON?

People living in prison are more at risk from infectious diseases than other people in the community.

Risk factors prior to prison

People in prison may be more likely to be exposed to infectious diseases or become seriously ill from these diseases for the following reasons:

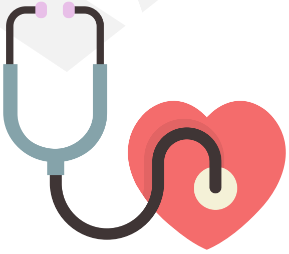

Underlying health conditions
People living in prison may have poorer general health when they come into prison than the average person of the same age outside prison. They may have pre-existing conditions such as heart problems, asthma, arthritis, cancer or diabetes which can make them more vulnerable to infections.

DRAFT

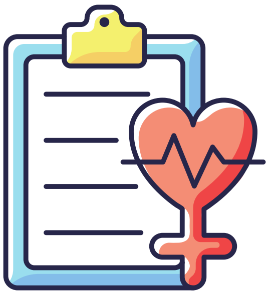

Other risk factors

People living in prison may have suffered periods of homelessness or rough sleeping which can make them more vulnerable to infection, and may have received limited access to healthcare and health information. Some people may also have been exposed to infection through unclean needles or unprotected sex.

The prison environment

The prison environment makes it easy for diseases to enter and spread, for the following reasons:

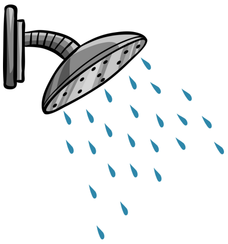

Sharing of cells, showers, toilets, and other communal spaces

Infectious diseases can easily spread through people using the same spaces and touching the same surfaces.

DRAFT

WHY IS VACCINATION SO IMPORTANT IN PRISON?

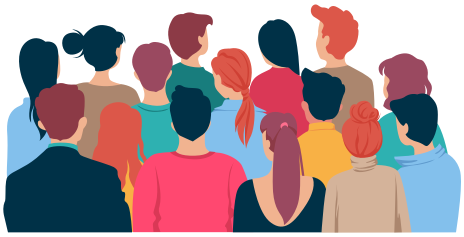

High number of people in an enclosed space

Prisons hold a lot of people and when you include staff that number increases. This makes it easier for infectious diseases to spread from one person to the next; and many prisons are not well ventilated.

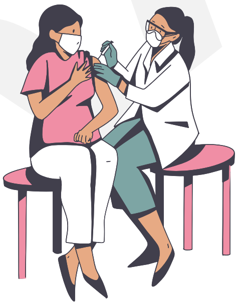

High turnover of people

People move from prison to prison as they serve their sentence. Staff also go in and out of prison. This flow of people through the prisons means there are many opportunities for infectious diseases to enter prisons.

DRAFT

HOW DOES VACCINATION WORK?

Vaccines work with your body's immune system to build protection against infection.

Vaccines reduce the risk of getting a disease by working with your body's immune system to build protection. When you get a vaccine, your immune system responds by:

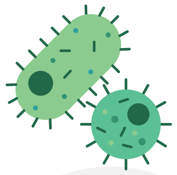

Recognizing the invading germ, such as the virus or bacteria

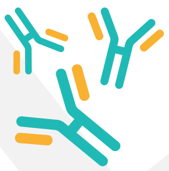

Producing antibodies which are produced naturally by the immune system to fight disease.

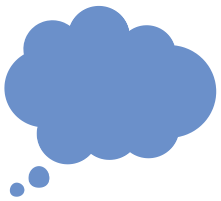

Remembering the disease and how to fight it so if you are exposed to the germ again, your immune system can quickly destroy it before you become unwell.

DRAFT

Our immune systems are designed to **remember**. Once exposed to one or more doses of a vaccine, we usually stay protected against a disease for years, decades or even a lifetime.

Rather than treating a disease after we get it, vaccines help stop us getting ill with the disease in the first place. It's much safer for your immune system to learn this through vaccination than by catching the diseases and treating them.

Vaccines are often given by injection but some can be given by mouth or sprayed into the nose.
Some vaccines are given in multiple doses, and it's important to have all the doses to be fully protected, whilst some require only one

DRAFT

HOW ARE VACCINES PRODUCED?

Like all medicines, every vaccine must go through very serious and robust testing to make sure it is safe and effective before it can be given

A new potential vaccine will only be tested on humans after it has gone through many rigorous safety tests in the lab, including animal testing.

Promising vaccines that are thought to be safe and effective are then tested in human volunteers. This is done in several phases, and at every stage the safety of the vaccine is assessed. People who participate in these trials are adequately informed and consent to their participation.

Doctors and independent scientists, as well as government health officials, inspect the results of these trials before vaccines are approved and licensed for use. A vaccine must be proven safe and effective before it will be approved. Vaccines are then monitored after being approved for the public.

DRAFT

Safety is assessed even after a vaccine has been fully licensed. Most countries have a system to feedback information about side effects. These records are used to check safety and make sure that vaccines continue to be as safe as possible.

Pharmaceutical companies, like all private companies, do make financial profit, but it is in their interest to produce safe vaccines which will protect people from disease.

The ingredients of a vaccine include:

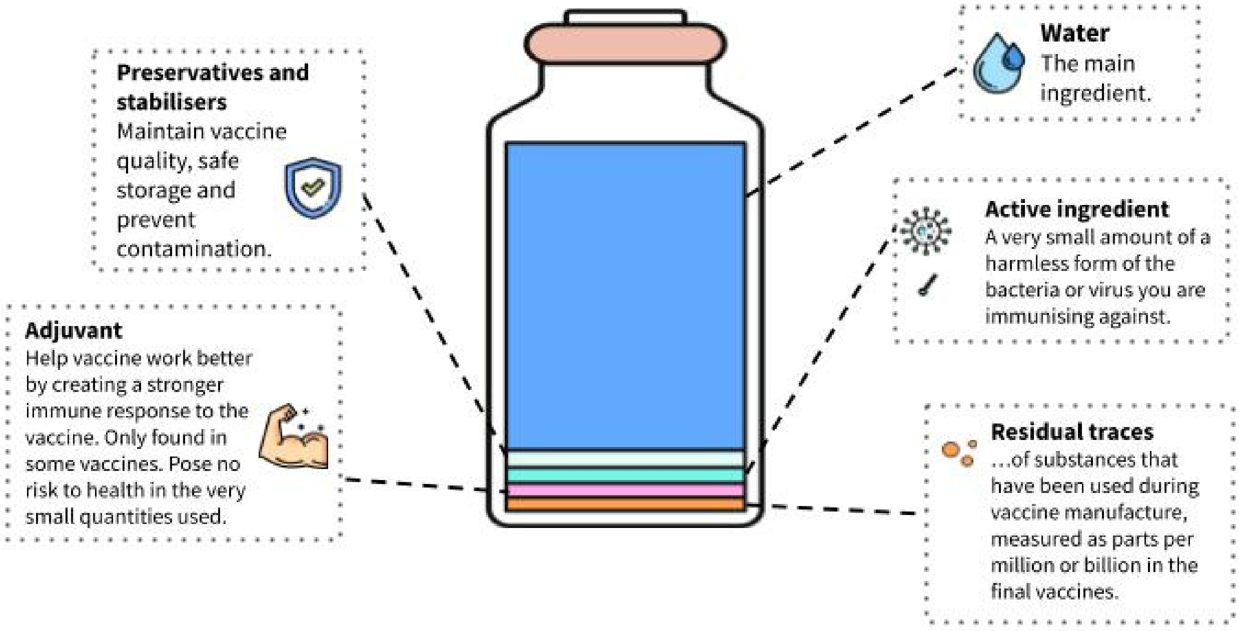

Some vaccines are included in the routine schedule and have been licensed for a very long time, for example the hepatitis B vaccine, whilst others are given in response to particular outbreaks or for groups that are particularly at risk, such as COVID-19 vaccination. COVID-19 vaccination was developed rapidly in response to a pandemic in which no one had protection from this new disease that was spreading rapidly, so there was great urgency. Therefore, more resources were made available and the vaccine was able to be produced ‘more quickly’. Different trial phases were run at the same time to help speed up the process and administrative barriers were removed, so things worked far quicker than usual, but approvals processes remained as important as ever.

DRAFT

SIDE EFFECTS

While vaccines can have side effects, most are mild and severe side effects are extremely rare.

All medicines can cause side effects, but vaccines are among the very safest. Research from around the world shows that vaccination is a very safe way to protect you, your family and your child's health.

Because of how vaccines work, it's always possible to have side effects after vaccination. These are often as a result of your body's immune system being activated to fight the vaccine as planned. Each person's body and immune system is different and that is why the reaction will be different for each person.

Most side effects are mild, such as a low-grade fever, or pain or redness at the injection site. You can use common painkillers such as paracetamol to manage your symptoms. Mild reactions go away within a few days on their own. Severe or long-lasting side effects are extremely rare. Any rare side effects that are discovered are investigated further.

DRAFT

Very rarely, people can have an allergic reaction soon after vaccination. This reaction may be a rash or itching affecting part or all of the body. The doctor or nurse giving the vaccine will know how to treat this. It does not mean that you should stop having vaccinations. Even more rarely, children or adults can have a severe reaction, within a few minutes of the vaccination, which causes breathing difficulties and can cause you or your child to collapse. This is called an anaphylactic reaction. A recent study has shown that there is only 1 anaphylactic reaction in about a million vaccinations. An anaphylactic reaction is a severe and immediate allergic reaction that needs urgent medical attention. The people who give vaccinations are trained to deal with anaphylactic reactions and children and adults recover completely with treatment.

You can always ask healthcare staff in prison about the side effects of any vaccine.

DRAFT

HOW EFFECTIVE VACCINES HAVE BEEN

Vaccines prevent up to 5 million deaths worldwide every year.

The story of polio vaccination

Do you know anyone with polio?

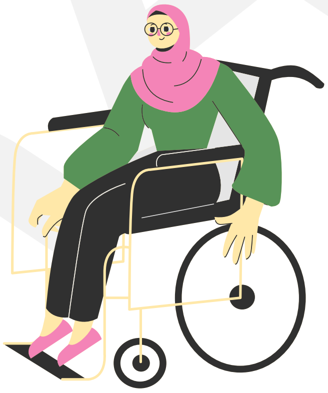

If not, this is probably because of vaccination. More than 1.5 million deaths in children have been avoided thanks to the polio vaccine, and more than 18 million people can walk today who would otherwise have been paralysed. Wild poliovirus cases have decreased by over 99% since 1988, from an estimated 350 000 cases in more than 125 endemic countries then, to 6 reported cases in 2021.

DRAFT

HOW EFFECTIVE VACCINES HAVE BEEN

Vaccination is a key part of primary health care. It reaches more people than any other health service worldwide. Vaccination currently prevents 3.5-5 million deaths every year from diseases like diphtheria, tetanus, pertussis, influenza and measles. Smallpox was a very deadly disease before the introduction of the smallpox vaccine. Since 1980, after a mass vaccination effort, there are no longer any cases.

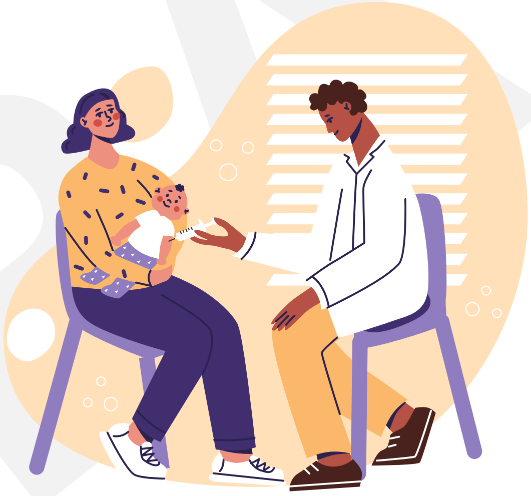

Since vaccines were introduced we have seen far fewer cases of diseases such as polio, which can cause serious illness, disability or even death. As these diseases become rarer, they become less visible. However, if people stop having vaccines, it's possible for serious infectious diseases that have become rare to quickly spread again.

DRAFT

VACCINES YOU MAY COME ACROSS IN PRISON

Hepatitis B - protecting against liver cancer or liver failure. Hepatitis B caused an estimated 820,000 deaths worldwide in 2019.

COVID-19 - protecting against severe disease, especially for older people or at risk adults with a weakened immune system

HPV - protecting against cervical cancer, penile cancer, head and neck cancer, and anal cancer. Approximately 95% of all cervical cancers are caused by HPV.

Influenza (Flu) - very important for risk groups (pregnant women, people with health conditions such as severe asthma, diabetes, having cancer treatment, those experiencing severe mental health difficulties, or severe learning disabilities) and to prevent prison outbreaks

DRAFT

VACCINES YOU MAY COME ACROSS IN PRISON

Tetanus, diphtheria and polio - protects against tetanus, diphtheria and polio, which can cause severe disability and death

Pneumococcal - protects against pneumonia and meningitis, which can cause severe disability and death

Meningococcal - protects against meningitis, which can cause severe disability and death

Measles, Mumps and Rubella (MMR) - protects against measles, mumps and rubella, which can cause severe disability and death

DRAFT

A FINAL WORD...

Vaccination is an important way of preventing infectious disease for all, but it is particularly important for people living in prison. People living in prison should have the right to access the same vaccinations as people in the community, and should have access to information about vaccines.

Vaccination helps protect your health and the health of those you come into contact with while in prison, as well as those you'll be in contact with when released.
Healthcare institutions have partnered with non-governmental organisations, academic institutions, former prisoners, prison services and government agencies across Europe to create materials that people living in prisons wanted regarding vaccines.

We hope that this brochure has been useful to you.

DRAFT

FURTHER QUESTIONS

If you have further questions about vaccination or your health, please discuss this with your healthcare staff in your prison.

If you'd like to know more about how we developed this brochure you can contact:

Engagement Team
Prison Reform Trust
15 Northburgh Street
London
EC1V 0JR

If you have any questions about the RISE-Vac project, please contact

Lara Tavoschi
University of Pisa
Lungarno Antonio Pacinotti, 43,
56126 Pisa
Italy

DRAFT
